# the impact of shared medical decision making on patient satisfaction in psychiatry

**DOI:** 10.1192/j.eurpsy.2022.1534

**Published:** 2022-09-01

**Authors:** K. Razki, Y. Zgueb, A. Aissa, U. Ouali, R. Jomli

**Affiliations:** Hôpital Razi, Service Psychiatrie A, La Manouba, Tunisia

**Keywords:** psychiatric care, shared medical decision making, the therapeutic process, the satisfaction

## Abstract

**Introduction:**

The era of the paternalisation of the patient is over, gradually giving way to new models, in particular that of “shared medical decision making”, with the aim of responding to the growing desires of the patient and giving priority to his autonomy.

**Objectives:**

to establish the influence of the new active position of the patient in the therapeutic process on the satisfaction of the psychiatric patient.

**Methods:**

This is a descriptive cross-sectional study that took place over a period of 5 months from April 2019 to August 2019 in two university hospital psychiatry departments of Razi Hospital in Tunisia . The questionnaire was administered outside any period of hospitalisation, in order to increase the reliability of responses. We used a pre-established form including socio-demographic data, clinical data concerning the patient’s mental disorder followed by a patient satisfaction questionnaire regarding the quality of care received in a psychiatric setting.

**Results:**

The patients interviewed in our study reported a good level of satisfaction (67.5%) with their involvement in the therapeutic process. However, 45.5% of the patients expressed dissatisfaction with the information provided to them by their doctor about their mental health status. The majority of the subjects surveyed expressed satisfaction with the quality of the interviews conducted during hospitalisation (71%) and with the time spent with the doctor (67%).

**Conclusions:**

Despite the fact that providing information to patients with mental health problems is a key element of patient satisfaction, not enough doctors actually include it in their daily practice.

**Disclosure:**

No significant relationships.

